# Trajectories and mental health-related predictors of perceived discrimination and stigma among homeless adults with mental illness

**DOI:** 10.1371/journal.pone.0229385

**Published:** 2020-02-27

**Authors:** Cilia Mejia-Lancheros, James Lachaud, Patricia O’Campo, Kathryn Wiens, Rosane Nisenbaum, Ri Wang, Stephen W. Hwang, Vicky Stergiopoulos

**Affiliations:** 1 MAP Centre for Urban Health Solutions, St Michael’s Hospital, Li Ka Shing Knowledge Institute, Toronto, ON, Canada; 2 Dalla Lana School of Public Health, University of Toronto, Toronto, ON, Canada; 3 Applied Health Research Centre, St Michael’s Hospital, Li Ka Shing Knowledge Institute, Toronto, ON, Canada; 4 Division of General Internal Medicine, Department of Medicine, University of Toronto, Toronto, ON, Canada; 5 Centre for Addiction and Mental Health, Toronto, ON, Canada; 6 Department of Psychiatry, University of Toronto, Toronto, ON, Canada; Maastricht University, NETHERLANDS

## Abstract

Stigma and discrimination toward individuals experiencing homelessness and mental disorders remain pervasive across societies. However, there are few longitudinal studies of stigma and discrimination among homeless adults with mental illness. This study aimed to identify the two-year group trajectories of stigma and discrimination and examine the predictive role of mental health characteristics among 414 homeless adults with mental illness participating in the extended follow-up phase of the Toronto At Home/Chez Soi (AH/CS) randomized trial site. Mental health-related perceived stigma and discrimination were measured at baseline, one, and two years using validated scales. Group-based-trajectory modelling was used to identify stigma and discrimination group trajectory memberships and the effect of the Housing First treatment (rent supplements and mental health support services) vs treatment as usual on these trajectories. The associations between mental health-related characteristics and trajectory group memberships were also assessed using multinomial logistic regression. Over two-years, three group trajectories of stigma and discrimination were identified. For discrimination, participants followed a low, moderate, or increasingly high discrimination group trajectory, while for stigma, participants followed a low, moderate or high stigma group trajectory. The Housing First treatment had no significant effect on discrimination or stigma trajectories groups. For the discrimination trajectories, major depressive episode, mood disorder with psychotic features, alcohol abuse, suicidality, severity of mental health symptoms, and substance use severity in the previous year were predictors of moderate and increasingly high discrimination trajectories. History of discrimination within healthcare setting was also positively associated with following a moderate or high discrimination trajectory. For the stigma trajectories, substance dependence, high mental health symptoms severity, substance use severity, and discrimination experiences within healthcare settings were the main predictors for the moderate trajectory group; while substance dependence, suicidality, mental health symptom severity, substance use severity and discrimination experiences within health care setting were also positive predictors for the high stigma trajectory group. Ethno-racial status modified the association between having a major depression episode, alcohol dependence, and the likelihood of being a member of the high stigma trajectory group. This study showed that adults experiencing mental illness and homelessness followed distinct stigma and discrimination group trajectories based on their mental health-problems. There is an urgent need to increase focus on strategies and policies to reduce stigma and discrimination in this population.

## Introduction

Stigma and discrimination are pervasive in many societies. Individuals often report feeling discriminated or stigmatized because of their economic situation, gender or sexual orientation, housing status, social position, ethno-racial status, mental health issues, and substance and alcohol use [1–7]. Furthermore, individuals with mental disorders face frequent stigma and discrimination even within social services and healthcare settings designed to support them [8–11]. Several stereotypes, including violent or dangerous behaviour, anti-social traits, and being responsible for their situation [12,13], and substance and alcohol use [6] have led to generalized stigma and discrimination against individuals with mental health problems.

Individuals with mental disorders are at a higher risk of homelessness, and a high proportion of individuals experiencing homelessness are also living with mental illness[14]. Homeless individuals are commonly stereotyped and discriminated against for their mental health status, substance and alcohol use, while also being blamed for their condition of being poor, homeless, unemployed, and for relying on social support benefits[11,15–17]. These experiences of stigma and discrimination happen in different settings, from everyday surroundings to healthcare, law enforcement, and social service environments [18,19]. In this population, stigma and discrimination constructs are not only attached to the state of homelessness but also to their other attributes including their health conditions (e.g., being HIV positive or having mental health problems)[20–22].

Stigma and discrimination can have a devastating impact on overall wellbeing, health and recovery of persons experiencing homelessness with and without co-occurring mental disorders[23]. Previous research suggests that discrimination is positively associated with higher emotional distress[24] and reduced social connections and group membership[25], while higher levels of internalized stigma are associated with worse mental health symptoms, such as depressive and psychotic symptoms, and suicidal ideation [26,27].

Limited research has examined longitudinal patterns of perceived stigma and discrimination among individuals who are homeless or have a mental illness[28]. The existing literature has mainly focused on single time point prevalence estimates, although longitudinal data is the only way to understand the persistence of, or changes in, experiences of stigma and discrimination over time. Moreover, while many studies focus on stereotypes and discrimination related to mental health in general, or specifically alcohol and substance use [6,7,29], there is a paucity of research on other mental disorders associated with persistent stigma and discrimination, either in the general population or among people experiencing homelessness. This limits our understanding of stigma and discrimination related to mental disorders, and therefore, the implementation of the policies and actions needed to reduce their persistence across settings.

In this study, we aimed to identify two-year group-based trajectories for mental health-related stigma and discrimination experienced by homeless adults with mental illness who participated in the Toronto site of the At Home/Chez Soi (AH/CS) randomized trial of Housing First with rent supplements and mental health support services. We also aimed to examine the predictive role of specific mental health diagnoses and problems on membership on these group trajectories. Finally, we tested the potential modifying effect of ethno-racial status on the association between mental health problems and discrimination and stigma group trajectories.

## Methods

### Study population and design

This study includes participants at the Toronto site of the AH/CS study, a large multi-site pragmatic randomized trial of Housing First (HF) services, conducted between 2009 and 2013 in 5 cities across Canada (Toronto, Moncton, Montreal, Winnipeg and Vancouver)[30]. Detailed information of the Toronto AH/CS study design has been published elsewhere [31]. Briefly, 575 participants were enrolled between October 2009 and July 2011. To be eligible they (i) were at least 18 years old; (ii) were absolutely homeless or precariously housed, with at least 2 episodes of absolute homelessness or one episode lasting over four weeks in the past year; and (iii) had a serious mental disorder with or without co-occurring alcohol or substance use disorder.

Participants were classified as having high needs (HN) for mental health support services if they exhibited low community functioning (<62 on the Multnomah Community Ability Scale), had psychotic or bipolar disorder, and met criteria for at least one of the following: two psychiatric hospitalizations in any one year in the past 5 years, comorbid alcohol or substance use disorder; or recent arrest or incarceration. The remaining participants were classified as having moderate needs (MN) for mental health support services [31]. HN and MN participants were randomized to receive either the HF treatment (rent supplements with assertive community treatment or intensive case management) or treatment as usual (TAU).

Participants were initially followed for two years between 2009 and 2013 (Phase I). Of the original 575 participants, 414 consented to additional follow-up from 2014 to 2017 (Phase II). This study includes Phase II participants, as stigma and discrimination were longitudinally assessed at yearly intervals over three-time points during this period. Of the 414 participants, 410 (99.0%) and 404 (97.6%) were included in the stigma and discrimination trajectory analyses because they contributed at least one data point during the data collection period.

### Ethics approval

The Toronto site of the AH/CS study received approval by the Research Ethics Board of St. Michael’s Hospital in Toronto, Canada. All study participants provided written informed consent to participate in both Phases I and II of the study. The AH/CS study is also registered with the International Standard Randomized Control Trial Number Register (ISRCTN42520374).

### Outcome measures

#### Discrimination

The ‘Unfair treatment” subscale of the Discrimination and Stigma Scale (DISC-12) developed by Thornicroft et al. [32] was used to measure perceived discrimination due to mental problems among our study participants. The scale was administered by face-to-face interviews at three-time points during Phase II of the AH/CS study: baseline, one-year, and two-year follow-up. The reference discrimination timeframe was set up to the previous six months from the administration date using the following question format: *“I would like to ask about times when you have been treated unfairly because of mental health problems in the last six months*. *There are 22 questions in this section*. *For each question*, *I will ask you to let me know whether each event has happened not at all [0]*, *a little [1]*, *moderately [2]*, *or a lot [3]”*. The mean score (range 0–3) was calculated as the sum of each item score (0, 1, 2, or 3) divided by the number of applicable and non-missing items[32]. Higher values indicate greater discrimination.

#### Stigma

The 10-item Stigma Experiences Scale [33] was used to assess perceived stigma experienced by our participants at baseline, one-year and two-years post-baseline of the AH/CS Phase II. During a face-to-face interview, the following introductory format was used for the administration of the scale: “*The next section asks about your own experiences with stigma*. *By stigma*, *we mean negative feelings people have toward people with a mental illness*. *In general*, *please tell me how often you think or feel about the following*: *[example] Do you think that people think less of you if they know you have a mental illness*?*”* The first two items were scored on a 5-point scale (never, rarely, sometimes, often, always), while the remaining items were scored on a 3-point scale (no, unsure, yes). The stigma index scores were calculated following the methodology used by Stuart H et al [33]. First, we dichotomized the original 5-point questions as 0 (never, rarely, sometimes) and 1 (often/always), and the 3-point scales as 0 (no, unsure) and 1 (yes). Next, the 0 and 1 values were summed into an overall count-based stigma index score ranging between 0 and 10 [33]. Higher values indicate a greater count of stigma experiences.

### Mental health measures

#### Mental disorders

The following mental health disorders were identified at baseline of the AH/CS Phase I based on DSM-IV criteria using the Mini International Neuropsychiatric Interview 6.0 [34]: Major Depressive Episode; Manic Episode or Hypomanic Episode; Posttraumatic Stress Disorder (PTSD); Panic Disorder; Mood Disorder with Psychotic Features; Psychotic Disorder; Alcohol and Substance Dependence Disorders; Alcohol Abuse, and Substance Abuse Disorders; and Suicidality.

#### Mental health symptom severity

The 14-question Colorado Symptom Index (CSI)[35,36] quantified the frequency of psychiatric symptoms. The overall summary CSI score ranged from 14 to 70 with lower scores indicating lower severity of mental illness.

#### Substance use severity

The first 5-items of the Substance Use Disorder Scale Short Screener (GAIN-SS score)[37] were used to assess substance use-related problems experienced in the previous year. GAIN scores range from 0 to 5, with higher values denoting greater severity of substance use problems.

#### Level of need for mental health services

HN and MN levels were based on the algorithm previously described in the study population and design.

#### Discrimination in health settings due to mental health problems

Participants were asked to report at baseline whether they had experienced discrimination within health setting due to their mental health problems in the previous year (no, yes). Discrimination was assessed using the following question: *“Of all the healthcare visit experiences in the last 12 months*, *have you ever felt that the doctor or healthcare staff you saw judged you unfairly or treated you with disrespect because of*: *your mental health issues*?”[38]

### Covariates

Age, gender and ethno-racial status were adjusted for in the analysis. Gender was dichotomized as men/women. Transgender and transsexual participants (n = 6) were included in the female category as their number was too small to carry out meaningful analyses as a separate category, and most self-identified as women. Furthermore, ethno-racial status (non-ethno-racial/ethno-racial) was also assessed as a modifying effect of mental health-related characteristics on the stigma and discrimination group trajectories membership. Since the present study was embedded within an HF randomized trial, the HF intervention group (HF treatment vs TAU) was included to adjust for any potential effect of the intervention on the estimates of the outcomes trajectories.

### Data analysis

We identified the stigma and discrimination trajectory groups using the Group-Based Trajectory Model framework[39–41] using the *traj s*tatistical program developed by Nagin et al[42,43].

Discrimination trajectories were estimated using a censored normal distribution model, as it a psychometric scale with clusters of values at the scales minimum and maximum. Stigma trajectories were estimated using the Zero-Inflated Poisson model (ZIP), as it is a count-based scale and to accommodate for the zero occurrences. We used the following steps to identify the discrimination and stigma trajectories memberships. First, we identified the number and shape of the group-based trajectories for both discrimination and stigma (unadjusted trajectory model) by fitting several models using the intercept (0), slope (1), quadratic (2) and cubic (3) functions. We selected the most suitable based-trajectory model for our participants by considering the following criteria as a whole [39]: (1) the Bayesian Information Criterion (BIC) (lower values indicate better fit); (2) the average values of the posterior probability of the assignment to the trajectory groups (> 0.70 for all groups is an indication that participants are well classified); (3) the weighted odds of correct classification into the corresponding trajectory group (values > 5 for all identified group indicate better assignment accuracy); (4) the confidence interval for the group membership probabilities (narrower confidence intervals indicates more accuracy of the estimated group probability).

Second, in order to adjust for the potential effect of the HF intervention on the observed trajectories, we added an indicator of the intervention group (HF vs TAU) in the final selected model. The trajectory groups derived from the adjusted models were used for subsequent analyses. We labelled low, moderate and high trajectory groups according to the group pathway participants followed.

For the discrimination group trajectories, we assigned the following labels: low, moderate, and increasing high. The ‘low trajectory group’ denotes the group of participants who had lower discrimination values at baseline and continued to have similar low values at the first and second year of follow-up. The ‘moderate trajectory group’ includes participants who had similar moderate mean discrimination values at baseline and 2-years follow-up, with slightly higher values at the 1-year of follow-up. The ‘increasing high trajectory group’ denotes the group of participants who started with higher discrimination mean values at baseline, had slightly lower values at 1 year of follow-up but had rising discrimination score values at the 2-year follow-up point.

Similarly, for the stigma group trajectories, we assigned the following labels: low, moderate, and high. The ‘low trajectory group’ denotes the group of participants who reported low values of stigma from baseline to the 2-year of the follow-up period. The ‘moderate trajectory group’ includes participants with similar moderate stigma values at baseline and year 2 follow up, with slightly decreased values at the 1-year of follow-up. The ‘high trajectory group’ denotes the group of participants who had persisting higher stigma values from baseline to the 2-year follow-up.

Third, for both outcomes, separate multinomial logistic models were used to evaluate the association between each mental health characteristic and group trajectory membership, adjusting for age, gender and ethno-racial group membership.

Finally, we explored the modifying effect of ethno-racial status by including a set of interaction terms between each mental health characteristic and ethno-racial status. We used the predictive margin effects (*margins* and *marginsplot* commands) to estimate the probability of our participants being a member of a discrimination and stigma trajectory group based on ethno-racial status and mental-health characteristics (this were only performed for those interactions that were statistically significant).

The Toronto AH/CS study was sampled to detect an effect size of 0.5 between HF and TAU groups for the major outcomes (housing stability, community functioning, quality of life), assuming statistical power of 80%[30,31,44]. For this secondary analysis, the statistical power remained >80% following a power calculation performed using the *power and sample-size features* in the Stata Software/SE 15.0 [45]. We also assumed a statistical significance level of 0.05 in our analyses. All the analyses were performed using Stata Software/SE 15.0 [45].

## Results

The description of baseline demographic and mental health characteristics of Phase II study participants (N = 414) are presented in S1 Table. Participants were on average 40.4 (±11.6) years old, were more often men (67.6%), and identified as having non-white ethno-racial status (56.8%). Substance dependence (39.6%), depression (36.5%), psychosis (35.3%), and alcohol dependence (30.7%) were the more prevalent mental disorders in our sample. Of our participants, 34.1% had a high level of need for mental health services, and 68.1% experienced suicidality.

### Discrimination group trajectories

**Fig 1:**
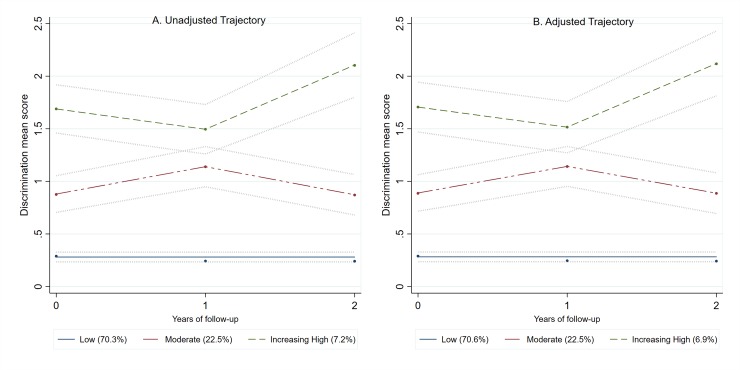
Unadjusted (A) and adjusted trajectory (B) membership for discrimination during the AH/CS Phase II Toronto Site study. Discrimination Trajectories description. Unadjusted Trajectory Model: Low, n = 295 (70.3%); Moderate, n = 88 (22.5%); Increasing High, n = 27 (7.2%). Trajectory model adjusted for Housing First intervention group: Low, n = 297 (70.6%); Moderate, n = 86 (22.4%); Increasing High, n = 27 (6.9%).

Fig 1 shows the unadjusted (panel A) and adjusted for HF intervention (panel B) trajectory groups for discrimination. The BIC criteria for the several groups and polynomial combination trajectories models are presented in S2 Table, and the growth functions and fitting estimates of the model growth are presented in S3 and S4 Tables, respectively. The HF intervention was not associated (S5 Table) with the probability of membership in any of the trajectory groups (Fig 1B). Both the unadjusted and adjusted group trajectory models (Fig 1) showed similar percentage of people within each trajectory group. In the adjusted trajectory groups, 70.6% of participants were more likely to be in the low discrimination trajectory, 22.4% in the moderate discrimination group trajectory, and approximately 6.9% in the increasing high discrimination trajectory group.

### Mental health characteristics and discrimination group trajectories

Table 1 summarizes the unadjusted and adjusted associations between mental-health characteristics and discrimination group trajectory. Participants with major depressive episodes were more likely to belong to the low discrimination trajectory group than moderate discrimination trajectory group. Participants with a mood disorder with psychotic symptoms or suicidality were significantly more likely to be a member of the moderate discrimination group than the low discrimination group. In contrast, participants with higher severity of mental health symptoms and those experiencing discrimination within health settings were more likely to be members of the moderate or high-perceived discrimination group. Participants with alcohol abuse were significantly more likely to be in the high discrimination trajectory group than in the low discrimination group. Ethno-racial status was not an effect modifier for discrimination trajectory groups.

**Table 1:** Associations (Relative Risk Ratio) between baseline mental-health related problems and discrimination trajectory membership during phase II of the AH/CS Toronto site study.

		Discrimination trajectory membership groups^a^			
		Multinomial logistic regression model			
N = 410		Moderate Trajectory (vs low trajectory)		Increasing High Trajectory (vs low trajectory)	
Mental health problem at baseline	N	RRR (95% CI)	p-value	RRR (95% CI)	p-value
**Major Depressive Episode (yes vs no)**					
Model 1^b^	410	0.52 (0.30–0.89)	0.018	1.04 (0.47–2.33)	0.311
Model 2^c^	410	0.53 (0.31–0.91)	0.022	1.17 (0.52–2.64)	0.711
Model 3^d^	410	0.54 (0.31–0.93)	0.025	1.18 (0.52–2.68)	0.693
**Manic Episode or Hypomanic Episode (yes vs no)**					
Model 1^b^	410	1.05 (0.50–2.23)	0.893	0.00 (0.0–00)	0.982
Model 2^c^	410	1.04 (0.48–2.21)	0.928	0.00 (0.0–00)	0.986
Model 3^d^	410	1.10 (0.51–2.38)	0.814	0.00 (0.0–00)	0.986
**PTSD (yes vs no)**					
Model 1^b^	410	1.33(0.77–2.29)	0.306	0.60 (0.20–1.79)	0.356
Model 2^c^	410	1.30(0.75–2.25)	0.342	0.58 (0.19–1.76)	0.335
Model 3^d^	410	1.36(0.78–2.37)	0.275	0.58 (0.19–1.80)	0.346
**Panic Disorder (yes vs no)**					
Model 1^b^	410	0.86 (0.43–1.71)	0.672	0.93 (0.31–2.80)	0.890
Model 2^c^	410	0.85 (0.43–1.70)	0.650	0.96 (0.31–2.94)	0.937
Model 3^d^	410	0.86 (0.43–1.72)	0.678	0.97 (0.31–3.00)	0.956
**Mood Disorder with Psychotic Features (yes vs no)**					
Model 1^b^	410	1.75 (1.02–3.00)	0.043	0.70 (0.23–2.11)	0.527
Model 2^c^	410	1.77 (1.03–3.05)	0.039	0.74 (0.24–2.24)	0.590
Model 3^d^	410	1.75 (1.01–3.02)	0.045	0.73 (0.24–2.22)	0.579
**Psychotic Disorder (yes vs no)**					
Model 1^b^	410	1.09 (0.66–1.81)	0.726	1.33 (0.60–2.98)	0.482
Model 2^c^	410	1.08 (0.65–1.79)	0.762	1.23 (0.54–2.79)	0.622
Model 3^d^	410	1.05 (0.63–1.75)	0.841	1.21 (0.53–2.79)	0.648
**Alcohol Dependence (yes vs no)**					
Model 1^b^	410	0.97 (0.57–1.63)	0.895	1.11 (0.48–2.57)	0.800
Model 2^c^	410	1.00 (0.59–1.70)	0.994	1.19 (0.51–2.81)	0.688
Model 3^d^	410	1.06 (0.61–1.85)	0.835	1.25 (0.51–3.06)	0.620
**Substance Dependence (yes vs no)**					
Model 1^b^	410	1.30 (0.80–2.12)	0.288	2.16 (0.97–4.77)	0.058
Model 2^c^	410	1.31 (0.80–2.14)	0.289	2.13 (0.95–4.78)	0.068
Model 3^d^	410	1.40 (0.84–2.33)	0.201	2.29 (0.99–5.29)	0.052
**Alcohol Abuse (yes vs no)**					
Model 1^b^	410	1.69 (0.87–3.28)	0.121	3.37 (1.37–8.30)	0.008
Model 2^c^	410	1.67 (0.86–3.27)	0.132	2.94 (1.16–7.45)	0.023
Model 3^d^	410	1.64 (0.84–3.22)	0.149	2.94 (1.15–7.51)	0.024
**Substance Abuse (yes vs no)**					
Model 1^b^	410	0.72 (0.29–1.80)	0.483	2.18 (0.77–6.22)	0.143
Model 2^c^	410	0.70 (0.28–1.75)	0.443	1.87 (0.64–5.43)	0.252
Model 3^d^	410	0.71 (0.28–1.78)	0.461	1.88 (0.64–5.48)	0.248
**Suicidality (yes vs no)**					
Model 1^b^	410	1.87 (1.07–3.29)	0.029	1.26 (0.53–2.98)	0.597
Model 2^c^	410	1.91 (1.09–3.37)	0.025	1.46 (0.60–3.53)	0.402
Model 3^d^	410	2.01 (1.13–3.56)	0.017	1.50 (0.61–3.66)	0.373
**High level of need for mental health services (high vs moderate)**					
Model 1^b^	410	0.76 (0.45–1.28)	0.305	1.28 (0.57–2.85)	0.553
Model 2^c^	410	0.76 (0.45–1.28)	0.304	1.24 (0.55–2.82)	0.604
Model 3^d^	410	0.78 (0.46–1.32)	0.351	1.27 (0.55–2.89)	0.576
**Mental Health symptom severity (Colorado Symptom Index score, range: 14–70)**					
Model 1^b^	398	1.03 (1.01–1.05)	0.004	1.04 (1.01–1.08)	0.020
Model 2^c^	398	1.03 (1.01–1.05)	0.001	1.05 (1.01–1.09)	0.011
Model 3^d^	398	1.03 (1.01–1.05)	0.002	1.05 (1.01–1.09)	0.010
**Substance use Severity in the previous year (GAIN score, range: 0–5)**					
Model 1^b^	392	1.12 (1.00–1.27)	0.054	1.23 (0.99–1.52)	0.056
Model 2^c^	392	1.14 (1.01–1.29)	0.041	1.23 (0.98–1.54)	0.072
Model 3^d^	392	1.17 (1.03–1.33)	0.018	1.25 (0.99–1.58)	0.060
**History of discrimination experiences in health settings due to mental health problems (yes vs no)**					
Model 1^b^	399	2.16 (1.32–3.53)	0.002	4.08 (1.70–9.78)	0.002
Model 2^c^	399	2.17 (1.32–3.54)	0.002	4.08 (1.68–9.93)	0.002
Model 3^d^	399	2.23 (1.36–3.66)	0.002	4.23 (1.72–10.37)	0.002

a. Adjusted trajectories for the HF intervention.

b. Unadjusted association

c. Adjusted for gender and age.

d. Adjusted for gender, age and ethno-racial status.

### Stigma trajectory group trajectories

**Fig 2:**
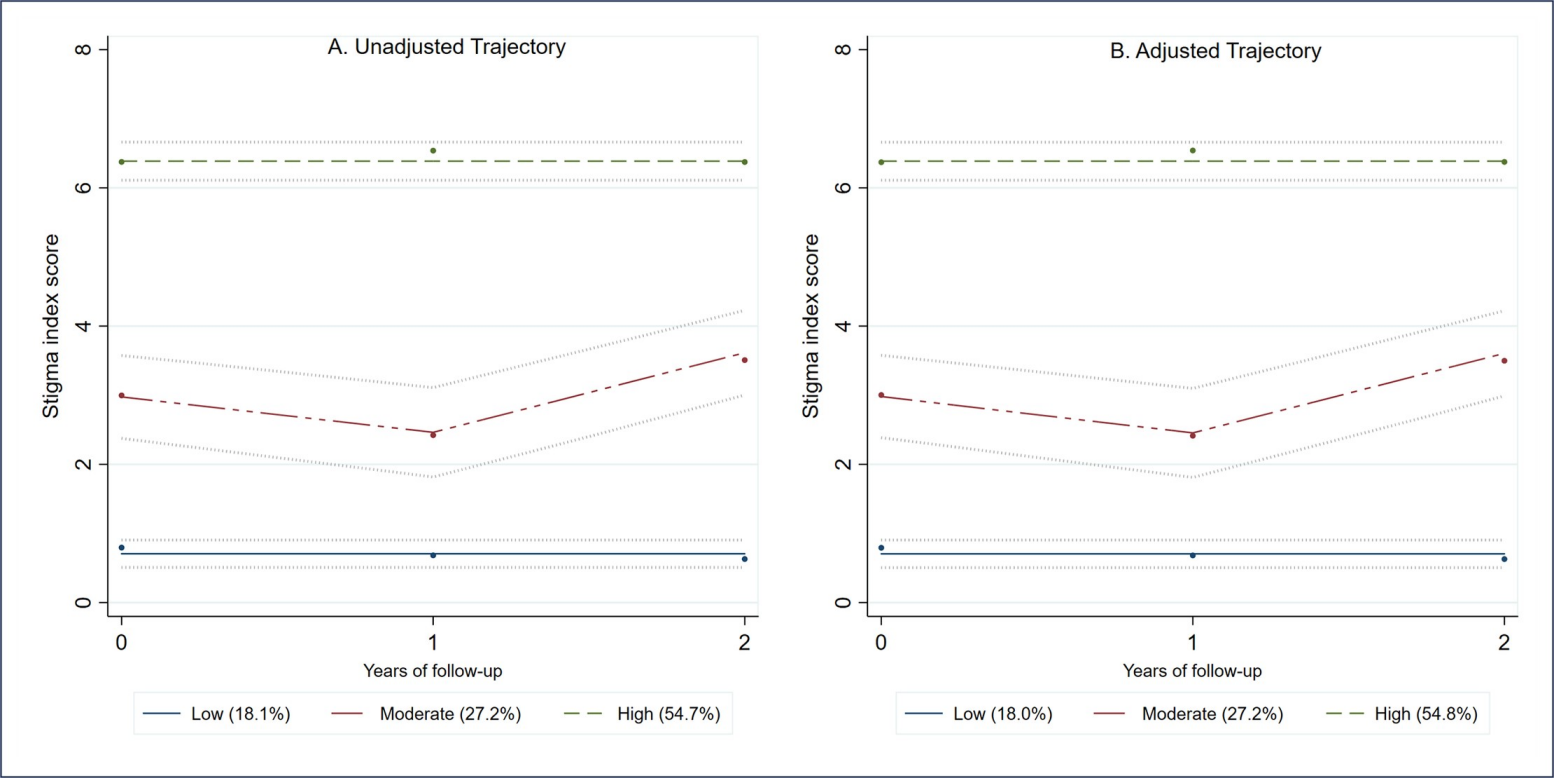
Unadjusted (A) and adjusted trajectory (B) membership for stigma during the AH/CS Phase II Toronto Site study. Stigma Trajectories description. Unadjusted Trajectory Model: Low, n = 80 (18.1%); Moderate, n = 103 (27.2%); High, n = 221(54.7%). Trajectory model adjusted for Housing First intervention group: Low, n = 81 (18.0%); Moderate, n = 105 (27.2%); High, n = 218(54.8%).

Fig 2 shows the unadjusted (Panel A) and adjusted for HF intervention (Panel B) trajectory groups for stigma. The BIC criteria for the group and polynomial combination trajectory models are shown in S6 Table, while the estimates of the growth parameters and those of the assignment and classification accuracy for the trajectory models are shown in S7 and S8 Tables, respectively. The adjustment of the stigma group trajectory for the HF intervention group showed a minimal and non-statistically significant effect on the trajectory membership probability values (S9 Table). Both the unadjusted and adjusted stigma trajectories models (Fig 2A and 2B) showed that the proportion of people in the stigma trajectory groups was similar for unadjusted and adjusted estimates. In the adjusted stigma trajectory model, 18.0% of participants were in the low stigma trajectory group, 27.2% of participants were more likely to be a member of the moderate stigma group, while over half (54.8%) of the participants were more likely to be in the high stigma trajectory group.

### Mental health characteristics and stigma group trajectories

Table 2 summarises the unadjusted and adjusted associations between mental health characteristics and stigma trajectory groups. Participants with substance dependence, higher mental health symptoms severity, higher substance use severity, and discrimination experiences within the health settings, were more likely to be in the moderate or high stigma trajectory group than in the low stigma trajectory group. Participants with suicidality were more likely to be members of the high stigma trajectory group than of the low stigma trajectory group. Participants with manic episode or hypomanic episode also tended to be part of the high stigma trajectory group, approaching but not reaching the statistical significance threshold (p = 0.077).

**Table 2:** Associations (Relative Risk Ratio) between baseline mental-health related problems and stigma trajectory membership during Phase II of the AH/CS Toronto site study.

		Stigma trajectory membership groups^a^			
		Multinomial logistic regression model			
N = 404		Moderate (vs low)		High (vs low)	
Mental health problem at baseline	N	RRR (95% CI)	p-value	RRR (95% CI)	p-value
**Major Depressive Episode (yes vs no)**					
Model 1^b^	404	1.15 (0.63–2.09)	0.651	1.06 (0.62–1.80)	0.829
Model 2^c^	404	1.13(0.62–2.07)	0.685	1.07 (0.63–1.83)	0.792
Model 3^d^	404	1.08 (0.59–1.98)	0.813	1.01 (0.59–1.73)	0.980
**Manic Episode or Hypomanic Episode (yes vs no)**					
Model 1^b^	404	1.25 (0.39–3.99)	0.702	2.43(0.91–6.49)	0.077
Model 2^c^	404	1.30 (0.41–4.32)	0.662	2.44 (0.91–6.55)	0.077
Model 3^d^	404	1.14 (0.35–3.68)	0.825	2.11 (0.78–5.75)	0.143
**PTSD (yes vs no)**					
Model 1^b^	404	2.00 (0.96–4.15)	0.064	1.64 (0.84–3.20)	0.149
Model 2^c^	404	2.05 (0.98–4.26)	0.056	1.63(0.83–3.18)	0.156
Model 3^d^	404	1.89 (0.90–3.97)	0.091	1.46(0.74–2.89)	0.272
**Panic Disorder (yes vs no)**					
Model 1^b^	404	0.99 (0.46–2.13)	0.980	0.79 (0.40–1.58)	0.511
Model 2^c^	404	1.01 (0.47–2.19)	0.974	0.79 (0.39–1.57)	0.500
Model 3^d^	404	0.98 (0.45–2.12)	0.950	0.75 (0.37–1.51)	0.419
**Mood Disorder with Psychotic Features (yes vs no)**					
Model 1^b^	404	1.55 (0.73–3.27)	0.251	164 (0.84–3.20)	0.149
Model 2^c^	404	1.53 (0.72–3.24)	0.264	1.65 (0.84–3.23)	0.144
Model 3^d^	404	1.60 (0.76–3.41)	0.219	1.75 (0.89–3.43)	0.106
**Psychotic Disorder (yes vs no)**					
Model 1^b^	404	0.87 (0.47–1.61)	0.651	1.03 (0.61–1.77)	0.903
Model 2^c^	404	0.86 (0.47–1.60)	0.643	1.03 (0.60–1.76)	0.918
Model 3^d^	404	0.93 (0.50–1.74)	0.822	1.14 (0.66–1.96)	0.649
**Alcohol Dependence (yes vs no)**	404				
Model 1^b^	404	1.99 (1.03–3.83)	0.041	1.62 (0.89–2.94)	0.113
Model 2^c^	404	1.93 (0.99–3.75)	0.054	1.67 (0.91–3.06)	0.096
Model 3^d^	404	1.72 (0.86–3.45)	0.125	1.42 (0.76–2.67)	0.272
**Substance Dependence (yes vs no)**	404				
Model 1^b^	404	3.05 (1.54–6.04)	0.001	3.66 (1.97–6.81)	<0.001
Model 2^c^	404	3.06 (1.54–6.08)	0.001	3.73 (2.00–6.97)	<0.001
Model 3^d^	404	2.86 (1.42–5.76)	0.003	3.44 (1.82–6.50)	<0.001
**Alcohol Abuse (yes vs no)**					
Model 1^b^	404	1.13 (0.46–2.79)	0.790	1.43 (0.65–3.13)	0.375
Model 2^c^	404	1.13 (0.45–2.80	0.796	1.41 (0.64–3.12)	0.392
Model 3^d^	404	1.21 (0.48–3.04)	0.681	1.55 (0.70–3.45)	0.284
**Substance Abuse (yes vs no)**					
Model 1^b^	404	2.08 (0.77–5.64)	0.148	1.06 (0.40–2.78)	0.910
Model 2^c^	404	2.16 (0.79–5.91)	0.135	1.01 (0.38–2.69)	0.948
Model 3^d^	404	2.06 (0.75–5.66)	0.163	0.99 (0.37–2.64)	0.978
**Suicidality (yes vs no)**					
Model 1^b^	404	1.58 (0.86–2.89)	0.138	1.80 (1.06–3.07)	0.030
Model 2^c^	404	1.57 (0.86–2.88)	0.143	1.88 (1.10–3.21)	0.020
Model 3^d^	404	1.47 (0.80–2.71)	0.217	1.73 (1.01–2.98)	0.046
**High level of need for mental health services (high vs moderate)**					
Model 1^b^	404	1.03 (0.55–1.92)	0.934	1.25 (0.72–2.16)	0.427
Model 2^c^	404	1.02 (0.54–1.90)	0.961	1.25 (0.72–2.17)	0.422
Model 3^d^	404	0.94 (0.50–1.78)	0.858	1.15 (0.66–2.01)	0.622
**Mental Health symptom severity (Colorado Symptom Index score, range: 14–70)**					
Model 1^b^	393	1.04 (1.01–1.06)	0.002	1.06 (1.04–1.08)	<0.001
Model 2^c^	393	1.04 (1.01–1.06)	0.003	1.06 (1.04–1.08)	<0.001
Model 3^d^	393	1.04 (1.01–1.06)	0.004	1.06 (1.04–1.08)	<0.001
**Substance use Severity in the previous year (GAIN score, range: 0–5)**					
Model 1^b^	387	1.28 (1.10–1.49)	0.001	1.26 (1.10–1.44)	0.001
Model 2^c^	387	1.28 (1.10–1.50)	0.002	1.28 (1.11–1.47)	0.001
Model 3^d^	387	1.26 (1.07–1.48)	0.005	1.24 (1.07–1.43)	0.004
**History of discrimination experiences in health settings due to mental health problems (yes vs no)**					
Model 1^b^	394	6.19 (2.58–14.83)	<0.001	11.08 (4.87–25.20)	<0.001
Model 2^c^	394	6.15 (2.56–14.77)	<0.001	11.12 (4.88–25.34)	<0.001
Model 3^d^	394	5.97 (2.48–14.35)	<0.001	10.72 (4.70–24.48)	<0.001

a. Adjusted trajectories for the HF intervention.

b. Unadjusted association.

c. Adjusted for gender and age.

d. Adjusted for gender, age and ethno-racial status.

**Fig 3:**
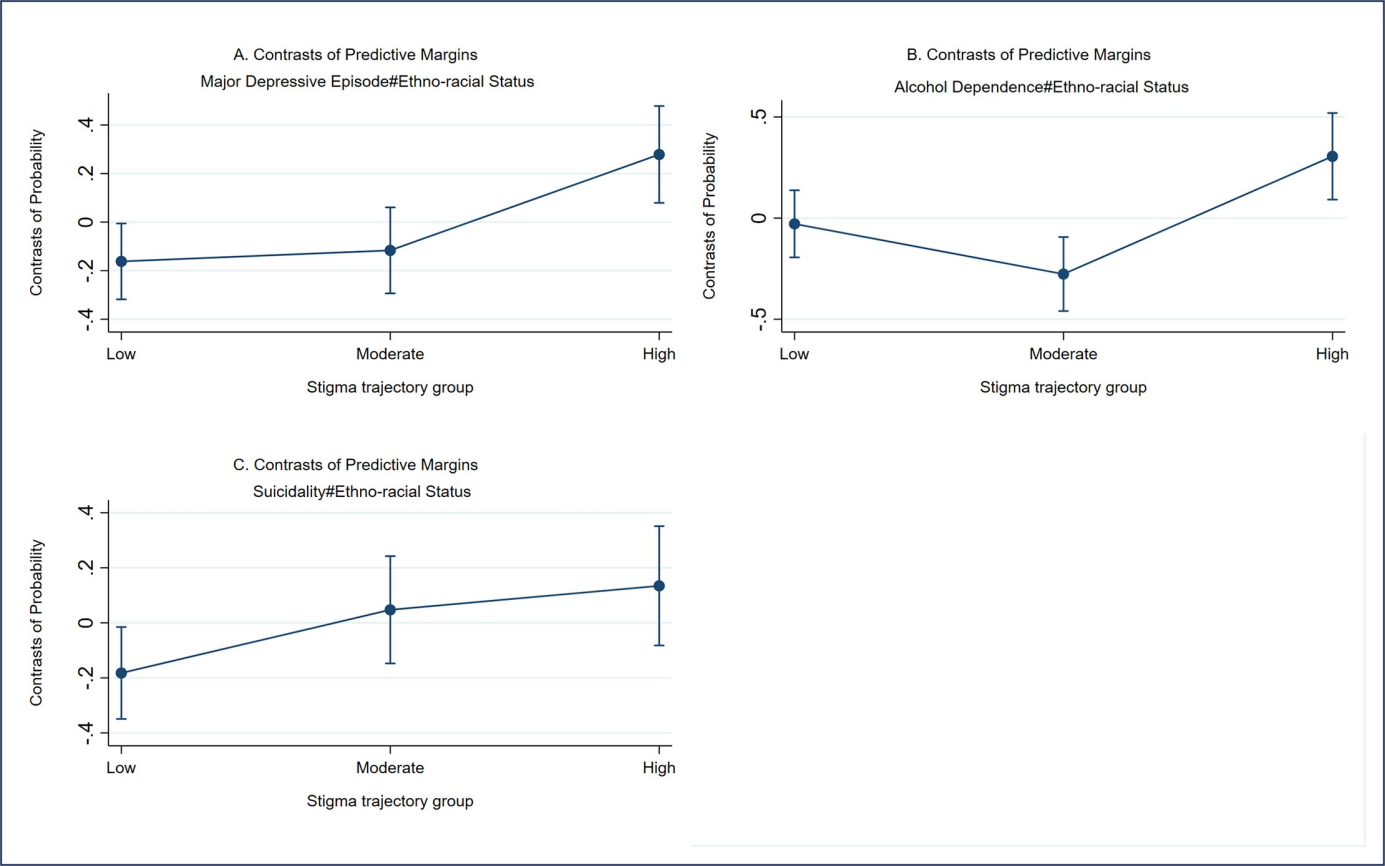
Contrast of the predictive margin interaction effect of a major depressive episode (A), alcohol dependence (B), Suicidality (C) with ethno-racial status on the stigma trajectory membership groups.

Ethno-racial status had a modifying effect on the associations between major depressive episodes, alcohol dependence or suicidality on stigma trajectory group membership. Specifically, participants with a major depressive episode or alcohol dependence who also identified as ethno-racial were more likely to be a member of the high stigma trajectory group (Fig 3A and 3B). Conversely, participants with suicidality who also identified as ethno-racial were more likely to be a member of the low stigma trajectory group (Fig 3C). There was no observed effect modifications of ethno-racial status for the other studied mental health characteristics.

## Discussion

In this two-year longitudinal cohort study of homeless adults with mental illness participating in Phase II of the AH/CS study at the Toronto site, we identified that our participants followed three distinct group trajectories for both discrimination (low, moderate, and increasing high) and stigma (low, moderate and high). We also found several baseline mental-health-related characteristics to predict discrimination and stigma trajectory group membership. For the stigma trajectory groups specifically, our study additionally identified an interaction between ethno-racial status and major depressive disorder, alcohol dependence, and suicidality.

The longitudinal trajectories analyses provide new information on perceived stigma and discrimination due to mental health problems among homeless adults with mental illness, showing that these attitudes and behaviours remain persistent over time, even within a more socially inclusive context as is Canada. A previous point in time analysis from Phase I of the AH/CS study showed that 23.8% of participants experienced discriminated due to their mental health problems, based on a different measuring tool, used to assess discrimination within the health care setting [38]. Similarly, Corrigan et al. found that around 19.1% of 696 people with mental health disabilities reported discrimination because of their mental disability, with 27.5% of these experiences occurring within the mental health system [46]. High levels of perceived stigma and discrimination toward people who are homeless and living with mental illness have also been previously reported in studies with cross-sectional design [15,17,47,48].

Our study also highlighted that receiving the HF intervention (rent supplements with assertive community treatment or intensive case management) did not influence the probability of membership in discrimination or stigma group trajectories. While the HF model has a positive impact on housing stability[49], these findings suggest the potential impact of HF on non-housing outcomes such as stigma and discrimination is limited. Therefore, multidimensional support services and efforts are needed [50] to address the structural socioeconomic barriers and mechanisms that contribute to stigma and of people experiencing or at risk of homelessness, with and without mental illness.

We found that several baseline clinical characteristics were significant predictors of participants’ membership to stigma and discrimination trajectory groups. In particular, we found that participants who had a major depressive episode at baseline were more likely to follow the low discrimination trajectory group. These findings may be explained by symptom resolution by the time of the Phase II study. Alternatively, depression might affect the perception and views of the nature and degree of everyday life circumstances [51,52], including discrimination experiences. Another potential explanation may be that depressive symptoms may be more acceptable compared to other mental disorders, possibly due to the high prevalence of depression in the general population, or to the passive acceptance or non-reactive or confrontational response by individuals with depression disorders [53].

Furthermore, we found that having a mood disorder with psychotic features, suicidality, and more severe mental health symptoms were associated with being a member of the moderate or high discrimination and stigma trajectory groups. Although death by suicide is quite common among homeless people [54–57], no previous studies have examined suicidality as a predictor of health-related discrimination and stigma trajectories in this population. Studies carried out in the general population or subgroups of people with mental illness and those from social minorities, have found general stigma and mental health-related stigma to be associated with suicidal ideation and high suicide risk [58–61]. Higher levels of prejudicial and stigmatizing attitudes have also been reported by individuals experiencing suicidal ideation or who have attempted suicide [59]. Mental illness severity has previously been identified as a predictor of stigma among people with mental health problems [62,63].

In our study, participants with alcohol abuse were also more likely to be a member of the high discrimination trajectory group, and those with substances dependence tended to be part of the moderate to high stigma group trajectory. In addition, when examining substance use severity, participants with higher severity scores were more likely to be a member of the moderate or high discrimination and stigma trajectory groups. Homeless people with alcohol and substance use disorders frequently experience generalized and health-related discrimination and stigma [12,64–66].

In addition, experiences of discrimination due to the mental health problems in a health care setting was a particularly strong predictor of membership to the high discrimination and stigma trajectory groups in our study population. Homeless people often feel unwelcome, misjudged and mistreated when visiting health care settings or attending health care encounters[67], reflecting pervasive discriminatory and stigmatizing attitudes and behaviours against the homeless population at the societal level.

Finally, we found that the participants’ ethno-racial status could modify the association between having a major depression episode or alcohol dependence and stigma trajectory group membership. Existing studies in non-homeless populations suggest that ethno-racial and cultural identities can contribute to variations in the perception of mental illness and the associated stigma[2,68]. Moreover, homeless individuals with a mental illness who identify as ethno-racially diverse not only face discrimination and stigma due to their homelessness state or mental health problems but also due to their skin colour[20], which can have a devastating impact on their recovery and overall wellbeing.

The following limitations should be noted when interpreting our study findings. First, we were only able to use two years of the follow-up data to estimate discrimination and stigma group trajectories. While this improves upon the one point in time measures in previous studies, the limited time points analysed may have reduced the variability and number of trajectories identified. Still, the final trajectories models were chosen based on the suggested criteria and methodology for this type of analysis [39,41–43]. Second, the identified discrimination and stigma group trajectories are flexible rather than fixed and individual pathways [39,40], and therefore their generalizability to other homeless populations may be limited. However, by accounting for the heterogeneity of the characteristics and features of the studied populations [35,36], these findings allow a closer understanding of the potential predictive factors of the longitudinal patterns of stigma and discrimination. Third, we used mental health-related factors that were assessed on average 2.8 years prior to the first discrimination and stigma measures, which may not represent mental health-related disorders and problems at the time when stigma and discrimination outcomes were measured. Finally, the number of transgendered and transsexual individuals was too small to analyze separately, and given that most self-identified as women, they were categorized with females in the analysis, foregoing the opportunity to explore their unique experiences.

The present study findings have the following research, practice, and policy implications. First, findings suggest that stigma and discrimination towards homeless people with mental illness are pervasive. Both social and health policy interventions are needed to reduce stigma and discrimination against vulnerable groups, including interventions within social services and health care settings. Our study also highlighted that clinical characteristics can predict the discrimination and stigma trajectories followed by homeless adults with mental illness. Therefore, efforts to reduce the detrimental effects of stigma and discrimination faced by adults experiencing homelessness with a mental illness may be targeted to particularly vulnerable subgroups [69,70]. For example, public anti-stigma and anti-discrimination educational campaigns that target specific population groups, such as students or health care providers [50,71,72], can contribute to increasing awareness and knowledge of mental health, alcohol and substance use problems, and homelessness. Interventions that centre around social-contact between people with and without mental illness have also been effective in improving stigma-related knowledge and attitude in a short term[72]; thus, it can also be a great strategy to reduce the stereotypes and discrimination against individuals who experience homelessness with a mental illness. Furthermore, systematic screening for experiences of stigma and discrimination could be implemented within social support and health-related service environments, in order to measurably reduce stereotyping and negative behaviours. Finally, given the persistently high levels of stigma and discrimination experienced by many vulnerable groups, further research on effective interventions is needed to impact policy and practice.

In conclusion, adults experiencing homelessness and mental illness face moderate to high trajectories of stigma and discrimination over time. Membership in each trajectory group can depend on specific mental health-related problems, such as major depressive episodes, suicidality or alcohol use. As such, there is a need to implement strategies and policies to reduce persisting and pervasive stigma and discrimination towards this population.

## Supporting information

S1 Table Baseline characteristic of study participants. (DOCX)

S2 Table BIC values for discrimination group-based trajectory model according to several groups and trajectory shapes. (DOCX)

S3 Table Model growth parameters for the unadjusted group-based discrimination trajectory and good classification and accuracy values. (DOCX)

S4 Table Model growth parameters for the adjusted group-based discrimination trajectory and good classification and accuracy values. (DOCX)

S5 Table The effect of Housing First on discrimination group-based trajectories probabilities. (DOCX)

S6 Table BIC values for stigma group-based trajectory model according to several groups and trajectory shapes. (DOCX)

S7 Table Model growth parameters for the unadjusted group-based stigma trajectory and good classification and accuracy values. (DOCX)

S8 Table Model growth parameters for the adjusted group-based stigma trajectory and good classification and accuracy values. (DOCX)

S9 Table The effect of Housing First on stigma group-based trajectories probabilities. (DOCX)
