# Supplementary material for: Trajectories and mental health-related predictors of perceived discrimination and stigma among homeless adults with mental illness
Source: PLoS One. 2020 Feb 27;15(2):e0229385. doi: 10.1371/journal.pone.0229385 (PMC7046214; doi:10.1371/journal.pone.0229385)
Supplement: S1 Table — (DOCX) [file pone.0229385.s001.docx]

**Table S1. Baseline characteristic of study participants.**

|  |  |  | |
| --- | --- | --- | --- |
| **N=414** | **N** | **%** | **Missing, n (%)** |
| **Demographic characteristics** |  |  |  |
| **Age (in years,** Mean ±SD) | **414** | 40.4(11.6) |  |
| **Gender** | **414** |  |  |
| Men | 280 | 67.6 |  |
| Women* | 134 | 32.4 |  |
| **Ethno-racial status** | **414** |  |  |
| No | 179 | 43.2 |  |
| Yes | 235 | 56.8 |  |
| **Mental health problems at baseline** |  |  |  |
| **Major Depressive Episode** | **414** |  |  |
| No | 263 | 63.5 |  |
| Yes | 151 | 36.5 |  |
| **Manic Episode or Hypomanic Episode** | **414** |  |  |
| No | 371 | 89.6 |  |
| Yes | 43 | 10.4 |  |
| **PTSD** | 410 |  |  |
| No | 319 | 77.1 |  |
| Yes | 95 | 22.9 |  |
| **Panic Disorder** | **414** |  |  |
| No | 351 | 84.8 |  |
| Yes | 63 | 15.2 |  |
| **Mood Disorder with Psychotic Features** | **414** |  |  |
| No | 325 | 78.5 |  |
| Yes | 89 | 21.5 |  |
| **Psychotic Disorder** | **414** |  |  |
| No | 268 | 64.7 |  |
| Yes | 146 | 35.3 |  |
| **Alcohol Dependence** | **414** |  |  |
| No | 287 | 69.3 |  |
| Yes | 127 | 30.7 |  |
| **Substance Dependence** | **414** |  |  |
| No | 250 | 60.4 |  |
| Yes | 164 | 39.6 |  |
| **Alcohol Abuse** | **414** |  |  |
| No | 357 | 86.2 |  |
| Yes | 57 | 13.8 |  |
| **Substance Abuse** | **414** |  |  |
| No | 375 | 90.6 |  |
| Yes | 39 | 9.4 |  |
| **Suicidality** | **414** |  |  |
| No | 132 | 31.9 |  |
| Yes | 282 | 68.1 |  |
| **Level of need for mental health services** | **414** |  |  |
| Moderate | 273 | 65.9 |  |
| High | 141 | 34.1 |  |
| **Mental health symptom severity**  **(Colorado Symptom Index score (range:14-70)),** Mean ±SD | **402** | 40.4(12.7) | 12(2.9) |
| **Substance use severity in the previous year (GAIN score, range: 0-5),** Mean ±SD | **392** | 4.0(2.5) | 18(4.4) |
| **Self-reported discrimination in health setting due to mental health problems** | **403** |  | 11(2.7) |
| No | 242 | 60.1 |  |
| Yes | 161 | 39.9 |  |
| **Housing First (HF) trial participants** | **414** |  |  |
| Treatment as usual | 175 | 42.3 |  |
| HF intervention | 239 | 57.7 |  |

*Women group included 6 transgendered /transsexual participants)
