# Supplementary material for: Trajectories and mental health-related predictors of perceived discrimination and stigma among homeless adults with mental illness
Source: PLoS One. 2020 Feb 27;15(2):e0229385. doi: 10.1371/journal.pone.0229385 (PMC7046214; doi:10.1371/journal.pone.0229385)
Supplement: S2 Table — (DOCX) [file pone.0229385.s002.docx]

**Table S2. BIC values for the discrimination Group-Based Trajectory Model according to several groups and trajectory shapes.**

| **Number of groups** | **Trajectory shapes** | **BIC^a^**  (Number of observations:1078) | **BIC^a^**  (Number of participants:410) |
| --- | --- | --- | --- |
| **2** | 0 0 | 1005.77 | -1003.83 |
| **2** | 0 1 | -1007.65 | -1005.23 |
| **2** | 0 2 | -1010.77 | -1007.87 |
| **2** | 1 1 | -1009.73 | -1006.83 |
| **2** | 1 2 | -1012.87 | -1009.49 |
| **2** | 2 2 | -1016.04 | -1012.17 |
| **3** | 0 0 0 | -996.50 | -991.80 |
| **3** | 0 1 1 | -997.61 | -993.74 |
| **3** | 0 1 2 | -999.19 | -994.84 |
| **3** | 0 2 2 | -997.31 | -992.48 |
| **3** | 0 2 0 | -997.38 | -993.34 |
| **3** | 1 1 0 | -1001.52 | -997.66 |
| **3** | 1 1 1 | -999.51 | -995.15 |
| **3** | 1 1 2 | -1001.10 | -996.26 |
| **3** | 1 2 0 | -999.05 | -994.70 |
| **3** | 1 2 1 | -1000.07 | -995.24 |
| **3** | 1 2 2 | -999.37 | -994.05 |
| **3** | 2 1 0 | -1001.18 | -996.83 |
| **3** | 2 1 1 | -1002.40 | -997.57 |
| **3** | 2 1 2 | -1003.96 | -998.65 |
| **3** | 2 2 0 | -1001.48 | -996.64 |
| **3** | 2 2 1 | -1002.46 | -997.10 |
| **3** | 2 2 2 | -1001.53 | -995.73 |
| **3** | 3 3 3* | -1012.00 | -1004.75 |
| **4** | 0 0 0 0 ** | -994.95 | -991.08 |

a. Bayesian information criterion

* Variance matrix was non-symmetric and the model has not fitted well the data.

** One group has very small observations, and variance matrix was non-symmetric.
