# Supplementary material for: Trajectories and mental health-related predictors of perceived discrimination and stigma among homeless adults with mental illness
Source: PLoS One. 2020 Feb 27;15(2):e0229385. doi: 10.1371/journal.pone.0229385 (PMC7046214; doi:10.1371/journal.pone.0229385)
Supplement: S3 Table — (DOCX) [file pone.0229385.s003.docx]

**Table S3. Model growth parameters for the unadjusted group-based discrimination trajectory and good classification and accuracy values.**

|  | **Model Growth Parameters (Standard Errors)^a^** | | |
| --- | --- | --- | --- |
|  | **Intercept** | **Slope** | **Quadratic** |
| **Discrimination Trajectory group** | Estimate  (Standard Error) | Estimate  (Standard Error) | Estimate  (Standard Error) |
| Low | 0.17(0.03) |  |  |
| Moderate | 0.88(0.07) | 0.53(0.17) | -0.27(0.09) |
| Increasing High | 1.69(0.11) | -0.60(0.28) | 0.40(0.14) |
|  |  |  |  |
| Sigma | 0.46(0.01) |  |  |
|  | **Parameters of good classification and accuracy** | | |
|  | **Average Posterior Probability** | **Weighted (posterior probability) Odds of correct classification** | |
| Low | 0.94 | 6.10 | |
| Moderate | 0.82 | 14.43 | |
| Increasing High | 0.91 | 133.55 | |

**a.** Bayesian information criterion (BIC) (N=410 participants): -992.48.
