# Supplementary material for: Trajectories and mental health-related predictors of perceived discrimination and stigma among homeless adults with mental illness
Source: PLoS One. 2020 Feb 27;15(2):e0229385. doi: 10.1371/journal.pone.0229385 (PMC7046214; doi:10.1371/journal.pone.0229385)
Supplement: S4 Table — (DOCX) [file pone.0229385.s004.docx]

**Table S4. Model growth parameters for the adjusted group-based discrimination trajectory and good classification and accuracy values.**

| **Discrimination** | **Model Growth Parameters (Standard Errors)^a^ adjusted for Housing First Intervention group^b^** | | |
| --- | --- | --- | --- |
| **Trajectory group** | **Intercept** | **Slope** | **Quadratic** |
|  | Estimate  (Standard Error) | Estimate  (Standard Error) | Estimate  (Standard Error) |
| **Low** | 0.17(0.03) |  |  |
| **Moderate** | 0.89(0.07) | 0.51(0.17) | -0.26(0.09) |
| **Increasing High** | 1.71(0.12) | -0.52(0.29) | 0.41(0.14) |
|  |  |  |  |
| Sigma | 0.46(0.01) |  |  |
|  | **Parameters of good classification and accuracy** | | |
|  | **Average Posterior Probability** | **Weighted (posterior probability) Odds of correct classification** |  |
| Low | 0.94 | 6.02 |  |
| Moderate | 0.82 | 15.71 |  |
| Increasing High | 0.90 | 126.6 |  |

a. Bayesian information criterion (BIC) (N=410 participants): -997.69

b. Housing First (HF) intervention group: HF treatment vs treatment as usual(TAU).
