# Supplementary material for: Trajectories and mental health-related predictors of perceived discrimination and stigma among homeless adults with mental illness
Source: PLoS One. 2020 Feb 27;15(2):e0229385. doi: 10.1371/journal.pone.0229385 (PMC7046214; doi:10.1371/journal.pone.0229385)
Supplement: S6 Table — (DOCX) [file pone.0229385.s006.docx]

**Table S6. BIC values for stigma Group-Based Trajectory Model according to several groups and trajectory shapes.**

| **Number of groups** | **Trajectory shapes** | **BIC^a^**  (Number of observation:1101) | **BIC^a^**  (Number of participants:404) |
| --- | --- | --- | --- |
| **2** | 0 0 | -2669.56 | -2667.55 |
| **2** | 0 1 | -2672.90 | -2670.42 |
| **2** | 0 2 | -2676.43 | -2673.42 |
| **2** | 1 1 | -2676.40 | -2673.39 |
| **2** | 1 2 | -2679.89 | -2676.38 |
| **2** | 2 2 | -2682.53 | -2678.51 |
| **3** | 0 0 0 | -2639.21 | -2636.20 |
| **3** | 0 1 1 | -2643.81 | -2639.80 |
| **3** | 0 1 2 | -2647.30 | -2642.79 |
| **3** | 0 2 2 | -2647.89 | -2642.88 |
| **3** | 0 2 1 | -2644.72 | -2640.21 |
| **3** | 0 2 0 | -2641.27 | -2637.26 |
| **3** | 1 1 0 | -2642.50 | -2638.49 |
| **3** | 1 1 1 | -2650.30 | -2644.34 |
| **3** | 1 1 2 | -2649.35 | -2644.34 |
| **3** | 1 2 0 | -2642.50 | -2638.49 |
| **3** | 1 2 1 | -2647.10 | -2642.09 |
| **3** | 1 2 2 | -2650.30 | -2644.79 |
| **3** | 2 1 0 | -2645.77 | -2641.26 |
| **3** | 2 1 1 | -2649.11 | -2644.10 |
| **3** | 2 1 2 | -2652.61 | -2647.09 |
| **3** | 2 2 0 | -2647.12 | -2642.10 |
| **3** | 2 2 1 | -2650.53 | -2645.02 |
| **3** | 2 2 2 | -2653.75 | -2647.73 |
| **3** | 3 3 3* | -2664.21 | -2656.74 |
| **4** | 0 0 0 0 ** | -2642.07 | -2638.06 |
| **4** | 0 0 0 1** | -1002.96 | -997.64 |

a. Bayesian information criterion

***** Variance matrix was non-symmetric and the model has not fitted well the data.

* * One group has very small observations and variance matrix was non-symmetric.
