# Supplementary material for: Trajectories and mental health-related predictors of perceived discrimination and stigma among homeless adults with mental illness
Source: PLoS One. 2020 Feb 27;15(2):e0229385. doi: 10.1371/journal.pone.0229385 (PMC7046214; doi:10.1371/journal.pone.0229385)
Supplement: S7 Table — (DOCX) [file pone.0229385.s007.docx]

**Table S7. Model growth parameters for the unadjusted group-based stigma trajectory and good classification and accuracy values.**

| **Stigma** | **Model Growth Parameters (Standard Errors)^a^** | | |
| --- | --- | --- | --- |
| **Trajectory group** | **Intercept** | **Slope** | **Quadratic** |
| Low | -0.27( 0.14) |  |  |
| Moderate | 1.17(0.10) | -0.48(0.27) | 0.29(0.13) |
| High | 1.93(0.02) |  |  |
|  |  |  |  |
| Alpha | -2.49(0.15) |  |  |
|  | **Parameters of good classification and accuracy** | | |
|  | **Average Posterior Probability** | **Weighted (posterior probability) Odds of correct classification** | |
| Low | 0.84 | 23.04 | |
| Moderate | 0.80 | 10.38 | |
| Increasing High | 0.92 | 9.63 | |

**a.** Bayesian information criterion (BIC) (N=404 participants): -2637.26
