# Supplementary material for: Trajectories and mental health-related predictors of perceived discrimination and stigma among homeless adults with mental illness
Source: PLoS One. 2020 Feb 27;15(2):e0229385. doi: 10.1371/journal.pone.0229385 (PMC7046214; doi:10.1371/journal.pone.0229385)
Supplement: S8 Table — (DOCX) [file pone.0229385.s008.docx]

**Table S8. Model growth parameters for the adjusted group-based stigma trajectory and good classification and accuracy values.**

| **Stigma** | **Model Growth Parameters (Standard Errors)^a^ adjusted for Housing First Intervention group^b^** | | |
| --- | --- | --- | --- |
| **Trajectory group** | **Intercept** | **Slope** | **Quadratic** |
| Low | -0.27(0.15) |  |  |
| Moderate | 1.17(0.10) | -0.48(0.27) | 0.30(0.13) |
| High | 1.93(0.02) |  |  |
|  |  |  |  |
| Alpha | -2.49(0.15) |  |  |
|  | **Parameters of good classification and accuracy** | | |
|  | **Average Posterior Probability** | **Weighted (posterior probability) Odds of correct classification** | |
| Low | 0.83 | 22.26 | |
| Moderate | 0.79 | 10.10 | |
| High | 0.93 | 10.55 | |

**a.** Bayesian information criterion (BIC) (N=404 participants): -2637.26.

b. Housing First (HF) intervention group: HF treatment vs treatment as usual(TAU).
