# Supplementary material for: Trajectories and mental health-related predictors of perceived discrimination and stigma among homeless adults with mental illness
Source: PLoS One. 2020 Feb 27;15(2):e0229385. doi: 10.1371/journal.pone.0229385 (PMC7046214; doi:10.1371/journal.pone.0229385)
Supplement: S9 Table — (DOCX) [file pone.0229385.s009.docx]

**Table S9. The effect of Housing First on stigma group-based trajectories probabilities.**

|  | **Effect of Housing First intervention on predicted stigma membership probability, AH/CS second phase, Toronto Site** | | | | |
| --- | --- | --- | --- | --- | --- |
|  | **Discrimination trajectory membership probability** | | | | |
| **N=404** | **Low** | **Moderate** |  | **Increasing High** |  |
|  | **Ref. group** | **Log-odds estimates (Standard error)^a^** | **P-value** | **Log-odds estimates (Standard error)^a^** | **P-value** |
| **Housing First treatment (vs TAU)** | --- | -0.05(0.40 ) | 0.897 | 0.08(0.31) | 0.801 |

a. Estimated via multinomial logistic regression using the the *traj* statistical program for Group-Based Trajectory analysis in stata software.
